# *Biophysics Reports*: focus on theoretical and technical advances

**DOI:** 10.1007/s41048-015-0010-3

**Published:** 2015-08-04

**Authors:** Tao Xu

**Affiliations:** Institute of Biophysics, Chinese Academy of Sciences, 15 Datun Road, Chaoyang District, Beijing, 100101 China

Welcome to *Biophysics Reports*, a peer-reviewed international journal in cooperation with SpringerOpen, one of the largest open-access publishers in the biological science field.

Biophysics continues to be a crucial driving force for biological and biomedical research. Our goal is to provide a highly visible forum for the dissemination of innovative theories, methods and protocols, and novel findings based on technological developments. The current publication formats include Methods, Protocols, Research Articles, Puncta, and Reviews/Minireviews.

Although the journal focuses on biophysics, we believe there is a growing need for integration across multiple disciplines to respond to biological and biomedical challenges. As such, this journal will address a broad audience of researchers in the life sciences working on technological developments including but not limited to biophysics, cell biology, immunology, membrane biology, redox biology, photobiology, neurobiology, oncology, biochemistry, biomechanics and mechanobiology, structural biology, biomedical imaging, electron microscopy, nuclear magnetic resonance spectroscopy, computational biology, bioinformatics, nanomedicine, genetics, model organisms, protein science, developmental biology, stem cells, omics, and biomedical big data. We also encourage authors to submit research articles that include data based on specific methods or techniques. Unlike the ‘methods sections’ of specialized journals, we place a strong emphasis on detailed technical information, rigorous validation, and practical relevance to the journal’s content. It is our aim to encourage interdisciplinary exchange of technical tool boxes.

We are proud to present our distinguished Editorial Board consisting of worldwide experts from multiple disciplines. Our Editorial Office ensures that we offer rigorous and rapid peer review, free submission/publication, rapid publication upon acceptance, and compliance with all open-access mandates. We would like to acknowledge all those who have worked tirelessly to ensure the successful launch of *Biophysics Reports*. With the journal *Nature Methods* as a model, we aim to work with scientific and publishing colleagues worldwide to establish *Biophysics Reports* as a high-quality, high-visibility, and high-impact international journal. We call upon our colleagues around the globe to join us in supporting *Biophysics Reports*, and making this journal a significant platform that promotes method and technique innovation.



Tao Xu

Editor-in-Chief, *Biophysics Reports*

Director, Institute of Biophysics,

Chinese Academy of Sciences

